# Individual factors increasing complexity of drug treatment—a narrative review

**DOI:** 10.1007/s00228-019-02818-7

**Published:** 2020-04-01

**Authors:** Steffen J. Schmidt, Viktoria S. Wurmbach, Anette Lampert, Simone Bernard, HIOPP-6 Consortium, Stefan Wilm, Achim Mortsiefer, Attila Altiner, Lisa Sparenberg, Joachim Szecsenyi, Frank Peters-Klimm, Petra Kaufmann-Kolle, Walter E. Haefeli, Hanna M. Seidling, Petra A. Thürmann

**Affiliations:** 1 Department of Clinical Pharmacology, School of Medicine, Faculty of Health, Witten/Herdecke University, Alfred-Herrhausen-Straße 50, 58448 Witten, Germany; 2 Department of Clinical Pharmacology and Pharmacoepidemiology, University of Heidelberg, Im Neuenheimer Feld 410, 69120 Heidelberg, Germany; 3 Cooperation Unit Clinical Pharmacy, University of Heidelberg, Im Neuenheimer Feld 410, 69120 Heidelberg, Germany; 4 Philipp Klee-Institute for Clinical Pharmacology, HELIOS Clinic Wuppertal, Heusnerstraße 40, 42283 Wuppertal, Germany

**Keywords:** Drug treatment, Administration error, Nonadherence, Narrative review, Complexity factor, Patient-centered

## Abstract

**Purpose:**

Complexity of drug treatment is known to be a risk factor for administration errors and nonadherence promoting higher healthcare costs, hospital admissions and increased mortality. Number of drugs and dose frequency are parameters often used to assess complexity related to the medication regimen. However, factors resulting from complex processes of care or arising from patient characteristics are only sporadically analyzed. Hence, the objective of this review is to give a comprehensive overview of relevant, patient-centered factors influencing complexity of drug treatment.

**Methods:**

A purposeful literature search was performed in MEDLINE to identify potential complexity factors relating to the prescribed drug (i.e. dosage forms or other product characteristics), the specific medication regimen (i.e. dosage schemes or additional instructions), specific patient characteristics and process characteristics. Factors were included if they were associated to administration errors, nonadherence and related adverse drug events detected in community dwelling adult patients.

**Results:**

Ninety-one influencing factors were identified: fourteen in “dosage forms”, five in “product characteristics”, twelve in “dosage schemes”, nine in “additional instructions”, thirty-one in “patient characteristics” and twenty in “process characteristics”.

**Conclusions:**

Although the findings are limited by the non-systematic search process and the heterogeneous results, the search shows the influence of many factors on the complexity of drug treatment. However, to evaluate their relevance for individual patients, prospective studies are necessary.

**Electronic supplementary material:**

The online version of this article (10.1007/s00228-019-02818-7) contains supplementary material, which is available to authorized users.

## Introduction

A general definition of complexity of drug treatment is missing so far. Previous studies typically focus on the medication regimen, and, thus, mostly consider the number of drugs and the frequency of administration [1, 2].

The most popular tool to quantify complexity is the Medication Regimen Complexity Index (MRCI). This score is based on literature and interdisciplinary expert opinions and considers 65 aspects of medication regimen complexity that are related to the dosage form, the dosage scheme or additional instructions for the application [3, 4]. It was already shown that medication regimen complexity causes administration errors and (non-)intentional nonadherence [3, 5, 6] with known consequences such as higher costs [7], increased hospital admissions [7] and mortality [8]. However, medication regimen complexity is only one part of complexity of drug treatment, as it does not only depend on the medication regimen but also on other factors such as demographic and clinical factors [4, 9]. The total number of drugs, especially polypharmacy—usually defined as the simultaneously use of five or more drugs [10]—automatically influences the number of factors increasing complexity of drug treatment. This emphasizes the relevance of complexity to the individual patient: in older adults, at least one in three patients is affected by polypharmacy, making this issue highly relevant to a majority of patients [11, 12]. Other risk factors at the patient level are, for example, impaired dexterity [13] and hospitalization [14]. Yet, such factors of complexity of drug treatment are only presented selectively [9, 15, 16] and, in general, none of the most commonly used tools assessing complexity addresses these patient-relevant aspects of complexity [17].

When taking the patients’ perspective, a broader concept of complexity than the commonly used medication regimen complexity is needed. This work therefore aims to give a comprehensive and theory-driven overview of relevant, patient-centered factors influencing complexity of drug treatment.

## Method

An undirected inductive literature search was conducted in MEDLINE via Pubmed by the authors VSW and SJS to identify factors potentially increasing complexity of drug treatment. This was based on the already known consequences of complexity, i.e. administration errors and nonadherence and therefore the search terms used were “adherence”, “administration error”, “administration errors”, “complex*”, “compliance”, “medication adherence [MeSH]”, “medication errors [MeSH]”, “nonadherence”, “non adherence”, “non compliance”, “noncompliance”, “nonpersistence”, “non persistence”, “patient compliance [MeSH]”, “persistence”, “treatment failure” and “treatment refusal [MeSH]”. In addition, the references of the identified literature were handsearched for further relevant literature. All the factors found in the literature were summarized in a mind map (please see supplement figure No. 1) [18]. Thereby six superordinate categories of factors potentially contributing to complexity of drug treatment (i.e. dosage forms, product characteristics, dosage schemes, additional instructions, patient characteristics and process characteristics) have emerged and the literature search was concluded when no further categories were found, because all factors identified could be assigned to one of them. An expert panel including VSW, SJS, AL and HMS discussed the relevance of each category, and, thus, these six categories were expected to cover all relevant factors that either directly increase complexity, such as drug and regimen-related aspects, or indirectly complexify drug treatment, such as characteristics of the medication process or of the patient himself. Conclusively, the six categories were confirmed by all authors as part of a workshop.

Based on the results of this introductory search, a non-systematic, purposeful search was performed in MEDLINE via Pubmed. As a starting point, the literature of the initial search and the references of the selected articles were searched. Additionally, search terms related to the defined six categories were used (please see supplement table No. 1) and combined with the search terms of the inductive search (please see above). The references of the identified literature as well as similar articles proposed by Pubmed were handsearched for further relevant factors. Literature found in the inductive search was also included in this literature search. Factors attributing to complexity were derived from studies and reviews that assessed drug handling and administration by adults in primary care (self-administration of drugs). In order to consider only relevant patient-level aspects, factors influencing complexity were included if administration errors, nonadherence or related adverse drug events were reported. If there were any indications in the literature of other factors that did not yet fulfill the inclusion criteria (e.g. no outpatient setting), MEDLINE was explicitly searched for this factor. Two researchers, VSW and SJS, worked on the six superordinate categories and reviewed each other’s factors as well as the underlying sources before including them. In case of uncertainties of inclusion or exclusion of a complexity factor, a decision was sought in discussion between the two researchers and three pharmacists (AL, SB and HMS) with experience in adherence research. The literature search was finished when a saturation of information was reached and no further complexity factors were identified.

In addition, the validated and frequently used MRCI [4] was evaluated to identify further complexity factors related to the medication regimen.

## Results

Ninety-one patient-centered influencing factors on the complexity of drug treatment were identified: fourteen relating to “dosage forms”, five relating to “product characteristics”, twelve relating to “dosage schemes”, nine relating to “additional instructions”, thirty-one relating to “patient characteristics" and twenty relating to “process characteristics”. Seventeen of these factors were not identified in the initial search, but were found in the items of the MRCI [4]: four relating to “dosage forms”, six relating to “dosage schemes” and seven relating to “additional instructions”.

### Dosage forms

Almost all dosage forms were described as predictive for administration errors or nonadherence in a specific context. The use of inhalers [19], injection devices [20–22], transdermal patches [23] and nasal preparations [24] and even of solid [25] or liquid [26] oral dosage forms was accompanied by administration errors. Furthermore, nonadherence was described for the use of ophthalmic preparations like eye drops [27], rectal preparations [28, 29] such as suppositories and enemas as well as dermatological preparations (Table 1) [30, 31].

**Table 1:** Drug dosage forms influencing the correct administration on the part of the patient

Dosage form	Described effect	Description/Example	References
Inhalers	Administration error	No breath holding or too slow inhalation	[19]
Injection devices (non-prefilled)	Administration error	No desinfection, air injection into vial or waiting before needle removal	[20]
Injection devices (prefilled)	Administration error	Resuspending insulin incorrectly or no removal of protection caps	[21, 22]
Transdermal patches	Administration error	Choosing wrong administration site or no removal of old patches	[23]
Nasal preparations	Administration error	Overdosing by administring too many drops	[24]
Solid oral dosage forms	Administration error	Intake with too little fluid or wrong tilt of head	[25]
Liquid oral dosage forms	Administration error	Over- or underdosing using different measurement tools (syringe, cup, teaspoon)	[26]
Ophthalmic preparations	Administration error	Touching the eye ball	[32]
	Nonadherence		[27]
Dosage forms associated with nonadherence			
Rectal preparations [28, 29]		Dermatological preparations [30, 31]	
Dosage forms extracted from MRCI [4]			
Solid dosage forms for oropharyngeal use	Liquid dosage forms for oropharyngeal use	Otological preparations	Vaginal preparations

### Product characteristics

Similar drug names [33] and drug appearances [34, 35] led to administration errors as well as the patient-unfriendly nature of solid [25] or liquid oral drugs [36, 37]. Moreover, packaging that was difficult to open reduced adherence (Table 2) [37, 38].

**Table 2:** Product characteristics of drugs and their packaging with impact on administration errors or adherence

Characteristic	Described effect	Description/Example	References
Similar drug names	Administration error	Administration of wrong drug	[33]
Similar drug appearance	Administration error	Administration of wrong drug	[34, 35]
Patient-unfriendly nature of solid oral dosage forms	Administration error	Inappropriate modification of dosage form due to form of tablets or surface material	[25]
Patient-unfriendly nature of liquid oral dosage forms	Administration error	Dosing error due to viscosity or modified administration due to unpleasant flavor	[36, 37]
Product characteristics associated with nonadherence			
Intricate packaging [37, 38]			

### Dosage schemes

An already frequently used measure for the complexity of drug treatment is the number of drugs concurrently used. For this type of complexity, the outcome on the part of the patient, namely nonadherence, has been frequently evaluated. Indeed, the risk of nonadherence increased with an increasing number of regularly used drugs [39–41]. Moreover, intake once a week [42], the number of daily intakes [28, 43, 44] and the number of drugs per intake [45] also influenced adherence. The timing of drug administration [46] and necessity of tablet splitting [47] was associated with a decrease in adherence (Table 3).

**Table 3:** Dosage schemes resulting in administration errors and/or nonadherence

Dosage scheme	Described effect	Description/Example	References
Once weekly administration	Administration error	Overdosing by using every day	[42]
	Nonadherence		[48]
Tablet splitting	Administration error	Splitting despite missing notches	[49]
	Nonadherence		[47]
Dosage schemes associated with nonadherence			
Total number of drugs [39–41]	Administration more than two times daily [28, 43, 44]	More than one drug concurrently [45]	Administration at lunch time [46]
Dosage schemes extracted from MRCI [4]			
Pro re nata (as needed) medication	Administration every two days or less frequently	Fixed dosing interval	Use of multiple doses concurrently
Different doses of the same active ingredient at different times of day	Variable dosing		

### Additional instructions

For some medicines, additional instructions for correct use are necessary. Following these instructions can be difficult, so low adherence was observed when medicines had to be taken at fixed times of the day [50]. Similarly, nonadherence was caused by deviations from instructions, especially when drug intake depended on meals (Table 4) [51].

**Table 4:** Additional instructions increasing complexity of drug treatment

Instructions associated with nonadherence			
Administration at fixed times of the day [50]		Meal-dependent administration [51, 52]	
Instructions extracted from MRCI [4]			
Increasing doses	Additional instructions	Disintegrating tablets, capsules and powders	Intake with advised liquid
Decreasing doses	Crushing tablets	Opening capsules	

### Patient characteristics

It is obvious that patients’ characteristics may have an impact on drug use, i.e. visual impairment, cognitive decline or incomprehension may result in incorrect application or omission of administration, the latter measured as nonadherence. Sociodemographic characteristics like some age groups (younger than 65 years and older than 84 years) [53–55], not being partnered or married [56] or not having support in drug handling [47] and female sex [57] were related to nonadherence. Similarly, different levels of education (low as well as high levels of education) [58, 59], poor numeracy [60] and low health literacy [15] were associated with nonadherence. Moreover, unemployment [31] and the income of the patient (low-class and middle-class income) [61, 62] affected adherence negatively along with a busy lifestyle [28] and the use of alcohol [31] or illicit drugs [63].

Health-related conditions like cognitive [61, 64] and physical [13, 65] limitations, especially swallowing difficulties [25], were also associated with reduced adherence and administration errors. Furthermore, a disease duration less than 10 years [58], an advanced disease [58], and the presence of comorbidities [39], in particular depression [16], contributed to nonadherence.

Patients’ experiences with side effects [66] or a lack of symptom control [61] and low satisfaction with health care [67] had shown to affect adherence. Likewise, patients were more often nonadherent if they expressed concerns about drug treatment [39] or felt stigmatized by the disease [66]. Similarly, further attitudes like low acceptance of the disease [58] and a lack of interest in drug treatment [68] reduced adherence, as well as otherwise lacking knowledge of disease and drug treatment [61]. Additionally, the use of alternative medicines [69] and a negative attitude towards drug treatment in general [70] influenced adherence negatively (Table 5).

**Table 5:** Patient characteristics associated with incorrect administration of drugs and nonadherence

Patient characteristics associated with nonadherence		
Sociodemographic characteristics		
Younger than 65 years [53, 55]	Older than 84 years [54]	No partner/spouse [56]
No support in drug handling [47]	Female sex [57]	Low education level [58]
High education level [59]	Poor numeracy skills [60]	Low health literacy [15]
Unemployment [31]	Low income [61]	Middle-class income [62]
Busy lifestyle [28]	Alcohol or illicit drug use [31, 63]	
Health-related conditions		
Cognitive impairment [61, 64]	Physical impairment [13, 65]	Swallowing difficulties [25]
Disease duration less than ten years [58]	Advanced disease [58]	One or more comorbidities [39]
Depression [16]		
Experiences		
Experienced side effects [66]	Lack of symptom control [61]	Low satisfaction with health care [67]
Attitude towards disease/therapy		
Concerns about drug treatment [39]	Feeling stigmatized by disease/drug treatment [66]	Low acceptance of disease [58]
Lack of interest in drug treatment [68]	Lack of knowledge regarding disease/drug treatment [61]	Negative attitude towards drug treatment [70]
Use of alternative medicines [69]		

### Process characteristics

Lack of training in dosage form use [19, 71], frequently changing prescriptions [72] or modifications of an existing medication regimen [5] led to more administration errors. Moreover, filling a pill box, which is often used to organize medication was a complex task that was prone to errors [73]. A simpler language in patient information leaflets reduced administration errors compared with the common language standard [74]. In general, therapy instructions must be formulated in a comprehensible way and should not vary between different physicians [72].

In addition to factors leading to administration errors, other factors showed their influence on adherence behavior. Product changes like each new prescription [62], frequent generic substitutions [75] and changes in tablet color or shape increased the incidence for nonadherence [76]. Moreover, if patients received their medication from several pharmacies [77], consulted several prescribing physicians at the same time [77], were never treated by a specialist [55] or were discharged from hospital [78], they also were less adherent. Similarly, the adherence was reduced by the supply of small package sizes [79], high costs for patients [62] or drug therapies lasting longer than 5 years [66]. Furthermore, less frequent control visits [80] or complex measurements such as self-measured blood glucose concentrations were associated with intentional nonadherence [81]. Moreover, a lack of information about the disease and drug treatment [66] along with no use of a medication schedule [82] had a negative effect on adherence (Table 6).

**Table 6:** Process characteristics resulting in administration errors or nonadherence

Characteristic	Described effect	Description/Example	References
Lack of training in dosage form use	Administration error	No gentle exhalation before inhalation or no breath holding	[19, 71]
Frequently changing prescriptions	Administration error	Drug confusion or forgetting about how to take drug	[72]
Changes in existing medication regimen	Administration error	Wrong reaction to physicians advice or self-monitoring results	[5]
Use of pill boxes	Administration error	Filling with wrong drug	[73]
Difficult language in patient information leaflet	Administration error	Wrong preparation and pen self-injection technique	[74]
Lack of comprehensibility and transparency of the instructions for drug treatment	Administration error	Drug confusion or forgetting about how to take drug	[72]
Process characteristics associated with nonadherence			
Product changes			
New prescription [62]	Frequent generic substitution [75]	Changes in tablet color or shape [76]	
Supply parameters			
Large number of pharmacies [77]	Large number of prescribing physicians [77]	No treatment by specialists [55]	
Hospital discharge [78]	Small package size supply [79]	High costs [62]	
Drug treatment for more than five years [66]			
Monitoring			
Low frequency of control visits to the physician [80]	Complex measurements (self-performed) [81]		
Information			
Lack of information about the disease/ drug treatment [66]	No use of medication schedule [82]		

## Discussion

Many influencing factors have been found across all six predefined categories, supporting the assumption that the entire medication process contributes to complexity of drug treatment. However, the number of factors found in each category varies. This may be since there actually are fewer factors in some categories than in others or that the categories are studied to a different extent.

In order to be applicable, some of the factors identified require further specifications. For example, there is a known relationship between complexity of drug treatment and the total number of drugs used [39–41]. However, the number of drugs examined for nonadherence differs from study to study. A usual cut-off for this parameter is the daily use of five or more drugs, which is a common definition of polypharmacy, too [10]. For this number of drugs, a reduced adherence could be shown [83].

Furthermore, it must be kept in mind that all the complexity factors were mostly examined independently and were not compared with each other. Thus, the extent of the influence of individual factors in a specific patient cannot be determined, and it is not yet possible to predict sufficiently whether and to what extent different factors influence each other or not. Additionally, the underlying patient samples differ in many characteristics, e.g. age, morbidities or social environment, as well as the settings studied. Therefore, transferability to an individual patient level is limited, and the applicability in the respective setting should be verified.

Moreover, some of the complexity factors found only indirectly affect complexity of drug treatment. For instance, an association between age and adherence has already been shown, but it has to be considered that age as a complexity factor implies other factors such as the number of comorbidities and consequently the total number of drugs used [11]. Therefore, the relevance of each individual factor should be assessed before use.

Previous reviews concentrated on certain patients, e.g. with specific diseases [84, 85] or of older age [86, 87]. Restricted to such conditions, these reviews may assess factors quantitatively, but their results need to be further summarized to provide an overview of all possible influencing factors. It should be noted that some factors were reported superficially, like “complex medication regimen” [84–86] without specifying what a complex medication regimen actually looked like. To assess reasons for complexity of drug treatment, these reviews therefore provide limited evidence. Furthermore, most of the reviews used adherence as the only outcome parameter [84–87]. However, administration errors show the problems of the patients with their medication as well. Reviews focusing on patients’ administration errors reported little about their causes, indicating more generic circumstances such as low health literacy, cognitive impairment or poor communication as reasons [88]. In contrast to previous reviews, this work summarizes many of the factors influencing complexity of drug treatment without the restrictions mentioned and provides a comprehensive, patient-centered overview, assessing both administration errors and nonadherence. As a side effect, many items listed in the MRCI were confirmed by further literature supporting their relevance.

However, this literature search led to heterogeneous results regarding the characteristics of some factors. Factors such as solid oral dosage forms [25] show little informative value because their characteristics are formulated too generally. For age [53–55], level of education [58, 59] and income [61, 62], the literature even shows contradictory characteristics. Moreover, for some factors, even their influence on the individual patient generally remains unclear. For example, the attribute “additional instructions” has already reported to potentially increase complexity [4] as well as to improve adherence under certain circumstances [89]. An assessment, especially of ambiguous factors, seems reasonable before those are used to assess complexity.

This review has several limitations; in particular, several methodological issues must be considered. First, the six categories of complexity factors were defined based on an unstructured literature search and an expert panel. Consequently, neither a structured literature search nor a formal consensus technique was applied. Nevertheless, all the complexity factors found could be applied to one of the categories, suggesting that they comprehensively cover all aspects of complexity of drug treatment. Second, MEDLINE was the only literature database that was searched, both for the introductory search leading to the definition of the categories and for the further literature search to identify complexity factors. Thus, relevant results, i.e. complexity factors, may be missed as other databases, such as CINAHL or PsycINFO, may refer to further factors and, accordingly, even further categories. However, the search in MEDLINE already led to a large number of relevant literature, thus leading to a large number of complexity factors. Moreover, saturation of information was observed during literature search.

## Conclusion

To the best of our knowledge, this review is the first, most comprehensive work of its kind. It demonstrates that complexity of drug treatment is not based exclusively on the medication regimen and suggests that multiple complexity factors must be considered when analyzing complexity of drug treatment. Based on these results, the patients’ perspective on the complexity factors must be examined to understand the reasons for the emergence of complexity of drug treatment and, thus, develop targeted measures to simplify drug treatment. In future projects, algorithms will be developed to consider all these complexity factors when analyzing complexity of drug treatment (Project HIOPP-6 [90]). Only if complexity of drug treatment can be assessed in a standardized way for the individual patient, tailored measures can be found to simplify drug treatment for the patient.

## Electronic supplementary material

ESM 1 (PDF 528 kb)

ESM 2 (PDF 23 kb)
