# Supplementary material for: Individual factors increasing complexity of drug treatment—a narrative review
Source: Eur J Clin Pharmacol. 2020 Apr 1;76(6):745–54. doi: 10.1007/s00228-019-02818-7 (PMC7239823; doi:10.1007/s00228-019-02818-7)
Supplement: Supplementary file 1 — (PDF 528 kb) [file 228_2019_2818_MOESM1_ESM.pdf]

Supplement Figure No. 1: Complexity of drug treatment (mind map)

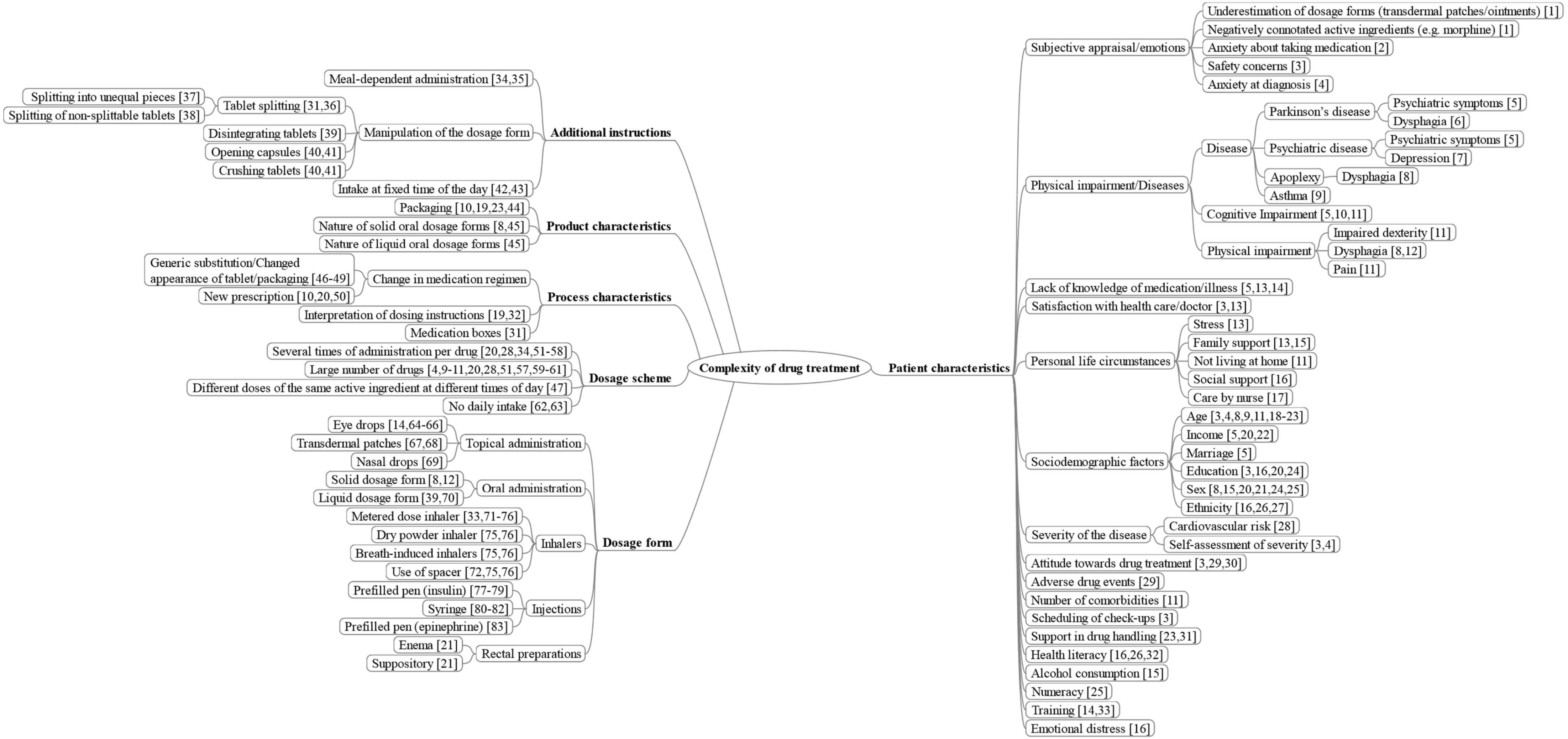

Created with FreeMind Version 1.0.1

Supplementary material to „Individual factors increasing complexity of drug treatment – a narrative review“ (in European Journal of Clinical Pharmacology), submitted by Steffen J. Schmidt*; Viktoria S. Wurmbach*; Anette Lampert; Simone Bernard; Walter E. Haefeli; Hanna M. Seidling; Petra A. Thürmann (*both authors contributed equally to the work)

Corresponding author: PD Dr. sc. hum. Hanna M. Seidling, University of Heidelberg, Department of Clinical Pharmacology and Pharmacoepidemiology, Cooperation Unit Clinical Pharmacy, Im Neuenheimer Feld 410, 69120 Heidelberg, Germany (E-mail: hanna.seidling@med.uni-heidelberg.de)

References

1. Radbruch L, Sabatowski R, Elsner F, Loick G, Kohnen N (2002) Patients' associations with regard to analgesic drugs and their forms for application -- a pilot study. Support Care Cancer 10 (6):480-485. doi:10.1007/s00520-002-0361-4
2. Schlesinger I, Rabinowitz D (2011) Medication phobia: a new cause of drug noncompliance in Parkinson disease. Clin Neuropharmacol 34 (6):220-223. doi:10.1097/WNF.0b013e3182372526
3. Bardel A, Wallander MA, Svardsudd K (2007) Factors associated with adherence to drug therapy: a population-based study. Eur J Clin Pharmacol 63 (3):307-314. doi:10.1007/s00228-006-0246-4
4. Nelson EC, Stason WB, Neutra RR, Solomon HS, McArdle PJ (1978) Impact of patient perceptions on compliance with treatment for hypertension. Med Care 16 (11):893-906. doi:10.1097/00005650-197811000-00001
5. Valldeoriola F, Coronell C, Pont C, Buongiorno MT, Camara A, Gaig C, Compta Y (2011) Socio-demographic and clinical factors influencing the adherence to treatment in Parkinson's disease: the ADHESON study. Eur J Neurol 18 (7):980-987. doi:10.1111/j.1468-1331.2010.03320.x
6. Fuh JL, Lee RC, Wang SJ, Lin CH, Wang PN, Chiang JH, Liu HC (1997) Swallowing difficulty in Parkinson's disease. Clin Neurol Neurosurg 99 (2):106-112. doi:10.1016/s0303-8467(97)00606-9
7. DiMatteo MR, Lepper HS, Croghan TW (2000) Depression is a risk factor for noncompliance with medical treatment: meta-analysis of the effects of anxiety and depression on patient adherence. Arch Intern Med 160 (14):2101-2107
8. Schiele JT, Quinzler R, Klimm HD, Pruszydlo MG, Haefeli WE (2013) Difficulties swallowing solid oral dosage forms in a general practice population: prevalence, causes, and relationship to dosage forms. Eur J Clin Pharmacol 69 (4):937-948. doi:10.1007/s00228-012-1417-0
9. Horne R, Weinman J (1999) Patients' beliefs about prescribed medicines and their role in adherence to treatment in chronic physical illness. J Psychosom Res 47 (6):555-567. doi:10.1016/s0022-3999(99)00057-4
10. Nikolaus T, Kruse W, Bach M, Specht-Leible N, Oster P, Schlierf G (1996) Elderly patients' problems with medication. An in-hospital and follow-up study. Eur J Clin Pharmacol 49 (4):255-259. doi:10.1007/bf00226324
11. Wimmer BC, Johnell K, Fastbom J, Wiese MD, Bell JS (2015) Factors associated with medication regimen complexity in older people: a cross-sectional population-based study. Eur J Clin Pharmacol 71 (9):1099-1108. doi:10.1007/s00228-015-1883-2
12. Schiele JT, Penner H, Schneider H, Quinzler R, Reich G, Wezler N, Micol W, Oster P, Haefeli WE (2015) Swallowing Tablets and Capsules Increases the Risk of Penetration and Aspiration in Patients with Stroke-Induced Dysphagia. Dysphagia 30 (5):571-582. doi:10.1007/s00455-015-9639-9
13. Morisky DE, Ang A, Krousel-Wood M, Ward HJ (2008) Predictive validity of a medication adherence measure in an outpatient setting. J Clin Hypertens (Greenwich) 10 (5):348-354
14. Ikeda H, Tsukamoto H, Sugimoto A, Sawa A, Crabtree BL, Byrd HJ, Murakami T, Mishima HK, Kihira K (2008) Clinical significance of topical instillation technique in Japanese glaucoma patients. Pharmazie 63 (1):81-85
15. Costa GL, Lamego RM, Colosimo EA, Valacio RA, Moreira Mda C (2012) Identifying potential predictors of high-quality oral anticoagulation assessed by time in therapeutic international normalized ratio range: a prospective, long-term, single-center, observational study. Clin Ther 34 (7):1511-1520. doi:10.1016/j.clinthera.2012.06.002
16. Kalichman SC, Ramachandran B, Catz S (1999) Adherence to combination antiretroviral therapies in HIV patients of low health literacy. J Gen Intern Med 14 (5):267-273. doi:10.1046/j.1525-1497.1999.00334.x
17. Corsonello A, Pedone C, Lattanzio F, Lucchetti M, Garasto S, Carbone C, Greco C, Fabbietti P, Incalzi RA (2009) Regimen complexity and medication nonadherence in elderly patients. Ther Clin Risk Manag 5 (1):209-216. doi:10.2147/tcrm.s4870
18. al Mahdy H, Seymour DG (1990) How much can elderly patients tell us about their medications? Postgrad Med J 66 (772):116-121. doi:10.1136/pgmj.66.772.116
19. Hurd PD, Butkovich SL (1986) Compliance problems and the older patient: assessing functional limitations. Drug Intell Clin Pharm 20 (3):228-231. doi:10.1177/106002808602000312
20. Kirkman MS, Rowan-Martin MT, Levin R, Fonseca VA, Schmittdiel JA, Herman WH, Aubert RE (2015) Determinants of adherence to diabetes medications: findings from a large pharmacy claims database. Diabetes Care 38 (4):604-609. doi:10.2337/dc14-2098
21. Boyle M, Ting A, Cury DB, Nanda K, Cheifetz AS, Moss A (2015) Adherence to Rectal Mesalamine in Patients with Ulcerative Colitis. Inflamm Bowel Dis 21 (12):2873-2878. doi:10.1097/mib.0000000000000562
22. Saeki H, Imafuku S, Abe M, Shintani Y, Onozuka D, Hagihara A, Katoh N, Murota H, Takeuchi S, Sugaya M, Tanioka M, Kaneko S, Masuda K, Hiragun T, Inomata N, Kitami Y, Tsunemi Y, Abe S, Kobayashi M, Morisky DE, Furue M (2015) Poor adherence to medication as assessed by the Morisky Medication

Adherence Scale-8 and low satisfaction with treatment in 237 psoriasis patients. J Dermatol 42 (4):367-372. doi:10.1111/1346-8138.12804
23. Sino CG, Sietzema M, Egberts TC, Schuurmans MJ (2014) Medication management capacity in relation to cognition and self-management skills in older people on polypharmacy. J Nutr Health Aging 18 (1):44-49. doi:10.1007/s12603-013-0359-2
24. Jankowska-Polanska B, Blicharska K, Uchmanowicz I, Morisky DE (2016) The influence of illness acceptance on the adherence to pharmacological and non-pharmacological therapy in patients with hypertension. Eur J Cardiovasc Nurs 15 (7):559-568. doi:10.1177/1474515115626878
25. Waldrop-Valverde D, Jones DL, Jayaweera D, Gonzalez P, Romero J, Ownby RL (2009) Gender differences in medication management capacity in HIV infection: the role of health literacy and numeracy. AIDS Behav 13 (1):46-52. doi:10.1007/s10461-008-9425-x
26. Osborn CY, Cavanaugh K, Wallston KA, Kripalani S, Elasy TA, Rothman RL, White RO (2011) Health literacy explains racial disparities in diabetes medication adherence. J Health Commun 16 Suppl 3:268-278. doi:10.1080/10810730.2011.604388
27. Trinacty CM, Adams AS, Soumerai SB, Zhang F, Meigs JB, Piette JD, Ross-Degnan D (2009) Racial differences in long-term adherence to oral antidiabetic drug therapy: a longitudinal cohort study. BMC Health Serv Res 9:24. doi:10.1186/1472-6963-9-24
28. Chapman RH, Benner JS, Petrilla AA, Tierce JC, Collins SR, Battleman DS, Schwartz JS (2005) Predictors of adherence with antihypertensive and lipid-lowering therapy. Arch Intern Med 165 (10):1147-1152. doi:10.1001/archinte.165.10.1147
29. Moss AC, Lillis Y, Edwards George JB, Choudhry NK, Berg AH, Cheifetz AS, Horowitz G, Leffler DA (2014) Attitudes to mesalamine questionnaire: a novel tool to predict mesalamine nonadherence in patients with IBD. Am J Gastroenterol 109 (12):1850-1855. doi:10.1038/ajg.2014.158
30. Grant RW, Devita NG, Singer DE, Meigs JB (2003) Polypharmacy and medication adherence in patients with type 2 diabetes. Diabetes Care 26 (5):1408-1412. doi:10.2337/diacare.26.5.1408
31. Lam PW, Lum CM, Leung MF (2007) Drug non-adherence and associated risk factors among Chinese geriatric patients in Hong Kong. Hong Kong Med J 13 (4):284-292
32. Davis TC, Wolf MS, Bass PF, 3rd, Thompson JA, Tilson HH, Neuberger M, Parker RM (2006) Literacy and misunderstanding prescription drug labels. Ann Intern Med 145 (12):887-894. doi:10.7326/0003-4819-145-12-200612190-00144
33. Giraud V, Roche N (2002) Misuse of corticosteroid metered-dose inhaler is associated with decreased asthma stability. Eur Respir J 19 (2):246-251. doi:10.1183/09031936.02.00218402
34. Stone VE, Hogan JW, Schuman P, Rompalo AM, Howard AA, Korkontzelou C, Smith DK, Hers S (2001) Antiretroviral regimen complexity, self-reported adherence, and HIV patients' understanding of their regimens: survey of women in the her study. J Acquir Immune Defic Syndr 28 (2):124-131. doi:10.1097/00042560-200110010-00003
35. Freigofas J, Haefeli WE, Schottker B, Brenner H, Quinzler R (2014) Indirect evidence for proton pump inhibitor failure in patients taking them independent of meals. Pharmacoepidemiol Drug Saf 23 (7):768-772. doi:10.1002/pds.3620
36. Hixson-Wallace JA, Dotson JB, Blakey SA (2001) Effect of regimen complexity on patient satisfaction and compliance with warfarin therapy. Clin Appl Thromb Hemost 7 (1):33-37
37. Freeman MK, White W, Iranikhah M (2012) Tablet splitting: a review of weight and content uniformity. Consult Pharm 27 (5):341-352. doi:10.4140/TCP.n.2012.341
38. Quinzler R, Gasse C, Schneider A, Kaufmann-Kolle P, Szecsenyi J, Haefeli WE (2006) The frequency of inappropriate tablet splitting in primary care. Eur J Clin Pharmacol 62 (12):1065-1073. doi:10.1007/s00228-006-0202-3
39. Bertsche T, Bertsche A, Krieg EM, Kunz N, Bergmann K, Hanke G, Hoppe-Tichy T, Ebinger F, Haefeli WE (2010) Prospective pilot intervention study to prevent medication errors in drugs administered to children by mouth or gastric tube: a programme for nurses, physicians and parents. Qual Saf Health Care 19 (5):e26. doi:10.1136/qshc.2009.033753
40. Kirkevold O, Engedal K (2010) What is the matter with crushing pills and opening capsules? Int J Nurs Pract 16 (1):81-85. doi:10.1111/j.1440-172X.2009.01814.x
41. Stubbs J, Haw C, Dickens G (2008) Dose form modification - a common but potentially hazardous practice. A literature review and study of medication administration to older psychiatric inpatients. Int Psychogeriatr 20 (3):616-627. doi:10.1017/s1041610207006047
42. Leopold NA, Polansky M, Hurka MR (2004) Drug adherence in Parkinson's disease. Mov Disord 19 (5):513-517. doi:10.1002/mds.20041
43. Hou JG, Wu LJ, Moore S, Ward C, York M, Atassi F, Fincher L, Nelson N, Sarwar A, Lai EC (2012) Assessment of appropriate medication administration for hospitalized patients with Parkinson's disease. Parkinsonism Relat Disord 18 (4):377-381. doi:10.1016/j.parkreldis.2011.12.007
44. Keram S, Williams ME (1988) Quantifying the ease or difficulty older persons experience in opening medication containers. J Am Geriatr Soc 36 (3):198-201. doi:10.1111/j.1532-5415.1988.tb01800.x

45. Liu F, Ranmal S, Batchelor HK, Orlu-Gul M, Ernest TB, Thomas IW, Flanagan T, Tuleu C (2014) Patient-centred pharmaceutical design to improve acceptability of medicines: similarities and differences in paediatric and geriatric populations. Drugs 74 (16):1871-1889. doi:10.1007/s40265-014-0297-2
46. Stange D, Kriston L, von Wolff A, Baehr M, Dartsch DC (2013) Medication complexity, prescription behaviour and patient adherence at the interface between ambulatory and stationary medical care. Eur J Clin Pharmacol 69 (3):573-580. doi:10.1007/s00228-012-1342-2
47. Lefkowitz M (2011) Do different body colors and labels of insulin pens enhance a patient's ability to correctly identify pens for injecting long-acting versus short-acting insulins? J Diabetes Sci Technol 5 (1):136-149. doi:10.1177/193229681100500119
48. Kesselheim AS, Bykov K, Avorn J, Tong A, Doherty M, Choudhry NK (2014) Burden of changes in pill appearance for patients receiving generic cardiovascular medications after myocardial infarction: cohort and nested case-control studies. Ann Intern Med 161 (2):96-103. doi:10.7326/m13-2381
49. Pruszydlo MG, Quinzler R, Kaltschmidt J, Haefeli WE (2008) [Medical problems and risks of switching drugs according to legal requirements of drug discount contracts in Germany]. Dtsch Med Wochenschr 133 (27):1423-1428. doi:10.1055/s-2008-1081094
50. Barber N, Parsons J, Clifford S, Darracott R, Horne R (2004) Patients' problems with new medication for chronic conditions. Qual Saf Health Care 13 (3):172-175. doi:10.1136/qhc.13.3.172
51. Gurwitz JH, Glynn RJ, Monane M, Everitt DE, Gilden D, Smith N, Avorn J (1993) Treatment for glaucoma: adherence by the elderly. Am J Public Health 83 (5):711-716. doi:10.2105/ajph.83.5.711
52. van Schayck CP, Bijl-Hofland ID, Folgering H, Cloosterman SG, Akkermans R, van den Elshout F, van Weel C (2002) Influence of two different inhalation devices on therapy compliance in asthmatic patients. Scand J Prim Health Care 20 (2):126-128. doi:10.1080/pri.20.2.126.128
53. Eldred LJ, Wu AW, Chaisson RE, Moore RD (1998) Adherence to antiretroviral and pneumocystis prophylaxis in HIV disease. J Acquir Immune Defic Syndr Hum Retrovirol 18 (2):117-125
54. Kruse W, Eggert-Kruse W, Rampmaier J, Runnebaum B, Weber E (1991) Dosage frequency and drug-compliance behaviour--a comparative study on compliance with a medication to be taken twice or four times daily. Eur J Clin Pharmacol 41 (6):589-592. doi:10.1007/BF00314990
55. Saini SD, Schoenfeld P, Kaulback K, Dubinsky MC (2009) Effect of medication dosing frequency on adherence in chronic diseases. Am J Manag Care 15 (6):e22-33
56. Claxton AJ, Cramer J, Pierce C (2001) A systematic review of the associations between dose regimens and medication compliance. Clin Ther 23 (8):1296-1310. doi:10.1016/s0149-2918(01)80109-0
57. Robin AL, Novack GD, Covert DW, Crockett RS, Marcic TS (2007) Adherence in glaucoma: objective measurements of once-daily and adjunctive medication use. Am J Ophthalmol 144 (4):533-540. doi:10.1016/j.ajo.2007.06.012
58. Libby AM, Fish DN, Hosokawa PW, Linnebur SA, Metz KR, Nair KV, Saseen JJ, Vande Griend JP, Vu SP, Hirsch JD (2013) Patient-level medication regimen complexity across populations with chronic disease. Clin Ther 35 (4):385-398 e381. doi:10.1016/j.clinthera.2013.02.019
59. Parkin DM, Henney CR, Quirk J, Crooks J (1976) Deviation from prescribed drug treatment after discharge from hospital. Br Med J 2 (6037):686-688. doi:10.1136/bmj.2.6037.686
60. Kuo SZ, Haftek M, Lai JC (2017) Factors Associated with Medication Non-adherence in Patients with End-Stage Liver Disease. Dig Dis Sci 62 (2):543-549. doi:10.1007/s10620-016-4391-z
61. Bangalore S, Kamalakkannan G, Parkar S, Messerli FH (2007) Fixed-dose combinations improve medication compliance: a meta-analysis. Am J Med 120 (8):713-719. doi:10.1016/j.amjmed.2006.08.033
62. Akarirmak U, Kocyigit H, Eskiyurt N, Esmaeilzadeh S, Kuru O, Yalcinkaya EY, Peker O, Ekim AA, Ozgirgin N, Calis M, Rezvani A, Cevikol A, Eyigor S, Sendur OF, Irdesel J, Group IS (2016) Influence of patient training on persistence, compliance, and tolerability of different dosing frequency regimens of bisphosphonate therapy: An observational study in Turkish patients with postmenopausal osteoporosis. Acta Orthop Traumatol Turc 50 (4):415-423. doi:10.1016/j.aott.2016.07.001
63. Moore TJ, Walsh CS, Cohen MR (2004) Reported medication errors associated with methotrexate. Am J Health Syst Pharm 61 (13):1380-1384
64. Stone JL, Robin AL, Novack GD, Covert DW, Cagle GD (2009) An objective evaluation of eyedrop instillation in patients with glaucoma. Arch Ophthalmol 127 (6):732-736. doi:10.1001/archophthalmol.2009.96
65. Taylor SA, Galbraith SM, Mills RP (2002) Causes of non-compliance with drug regimens in glaucoma patients: a qualitative study. J Ocul Pharmacol Ther 18 (5):401-409. doi:10.1089/10807680260362687
66. Tsai JC, McClure CA, Ramos SE, Schlundt DG, Pichert JW (2003) Compliance barriers in glaucoma: a systematic classification. J Glaucoma 12 (5):393-398
67. Lovborg H, Holmlund M, Hagg S (2014) Medication errors related to transdermal opioid patches: lessons from a regional incident reporting system. BMC Pharmacol Toxicol 15:31. doi:10.1186/2050-6511-15-31
68. Lampert A, Seiberth J, Haefeli WE, Seidling HM (2014) A systematic review of medication administration errors with transdermal patches. Expert Opin Drug Saf 13 (8):1101-1114. doi:10.1517/14740338.2014.926888

69. Patel RS, McGarry GW (2001) Most patients overdose on topical nasal corticosteroid drops: an accurate delivery device is required. J Laryngol Otol 115 (8):633-635
70. Ryu GS, Lee YJ (2012) Analysis of liquid medication dose errors made by patients and caregivers using alternative measuring devices. J Manag Care Pharm 18 (6):439-445. doi:10.18553/jmcp.2012.18.6.439
71. Dahl R, Backer V, Ollgaard B, Gerken F, Kesten S (2003) Assessment of patient performance of the HandiHaler compared with the metered dose inhaler four weeks after instruction. Respir Med 97 (10):1126-1133. doi:10.1016/s0954-6111(03)00162-8
72. Jones V, Fernandez C, Diggory P (1999) A comparison of large volume spacer, breath-activated and dry powder inhalers in older people. Age Ageing 28 (5):481-484. doi:10.1093/ageing/28.5.481
73. Crompton GK (1990) The adult patient's difficulties with inhalers. Lung 168 Suppl:658-662
74. Brocklebank D, Ram F, Wright J, Barry P, Cates C, Davies L, Douglas G, Muers M, Smith D, White J (2001) Comparison of the effectiveness of inhaler devices in asthma and chronic obstructive airways disease: a systematic review of the literature. Health Technol Assess 5 (26):1-149
75. Sanchis J, Gich I, Pedersen S, Aerosol Drug Management Improvement T (2016) Systematic Review of Errors in Inhaler Use: Has Patient Technique Improved Over Time? Chest 150 (2):394-406. doi:10.1016/j.chest.2016.03.041
76. Rau JL (2006) Practical problems with aerosol therapy in COPD. Respir Care 51 (2):158-172
77. Jehle PM, Micheler C, Jehle DR, Breitig D, Boehm BO (1999) Inadequate suspension of neutral protamine Hagendorn (NPH) insulin in pens. Lancet 354 (9190):1604-1607. doi:10.1016/S0140-6736(98)12459-5
78. Brown A, Steel JM, Duncan C, Duncan A, McBain AM (2004) An assessment of the adequacy of suspension of insulin in pen injectors. Diabet Med 21 (6):604-608. doi:10.1111/j.1464-5491.2004.01206.x
79. De Coninck C, Frid A, Gaspar R, Hicks D, Hirsch L, Kreugel G, Liersch J, Letondeur C, Sauvanet JP, Tubiana N, Strauss K (2010) Results and analysis of the 2008-2009 Insulin Injection Technique Questionnaire survey. J Diabetes 2 (3):168-179. doi:10.1111/j.1753-0407.2010.00077.x
80. Buysman E, Conner C, Aagren M, Bouchard J, Liu F (2011) Adherence and persistence to a regimen of basal insulin in a pre-filled pen compared to vial/syringe in insulin-naive patients with type 2 diabetes. Curr Med Res Opin 27 (9):1709-1717. doi:10.1185/03007995.2011.598500
81. Slabaugh SL, Bouchard JR, Li Y, Baltz JC, Meah YA, Moretz DC (2015) Characteristics Relating to Adherence and Persistence to Basal Insulin Regimens Among Elderly Insulin-Naive Patients with Type 2 Diabetes: Pre-Filled Pens versus Vials/Syringes. Adv Ther 32 (12):1206-1221. doi:10.1007/s12325-015-0266-5
82. Anderson BJ, Redondo MJ (2011) What can we learn from patient-reported outcomes of insulin pen devices? J Diabetes Sci Technol 5 (6):1563-1571. doi:10.1177/193229681100500633
83. Huang SW (1998) A survey of Epi-PEN use in patients with a history of anaphylaxis. J Allergy Clin Immunol 102 (3):525-526. doi:10.1016/s0091-6749(98)70145-9
