# Supplementary material for: Individual factors increasing complexity of drug treatment—a narrative review
Source: Eur J Clin Pharmacol. 2020 Apr 1;76(6):745–54. doi: 10.1007/s00228-019-02818-7 (PMC7239823; doi:10.1007/s00228-019-02818-7)
Supplement: Supplementary file 2 — (PDF 23 kb) [file 228_2019_2818_MOESM2_ESM.pdf]

Supplement Table No. 1: Search Terms

Category	*Search Terms*
Dosage forms	dosage form(s)<br>dosage forms [MeSH]<br>drug administration routes [MeSH]<br>drug delivery systems [MeSH]<br>equipment design [MeSH]<br>pharmaceutical form<br>pharmaceutical preparations [MeSH]
Product characteristics	drug compounding [MeSH]<br>drug design [MeSH]<br>drug formulation<br>drug labeling [MeSH]<br>drug packaging [MeSH]<br>product packaging [MeSH]<br>therapeutic equivalency [MeSH]
Dosage schemes	administration schedule<br>dosage schedule<br>dosage scheme(s)<br>dosing
Additional instructions	advice<br>direction(s)<br>information<br>instruction(s)
Patient characteristics	capacity<br>health conditions<br>health literacy<br>impairment<br>medication management capacity<br>patient satisfaction [MeSH]
Process characteristics	drug prescriptions [MeSH]<br>drugs, generic [MeSH]<br>patient discharge [MeSH]

Supplementary material to “Individual factors increasing complexity of drug treatment – a narrative review” (in European Journal of Clinical Pharmacology), submitted by Steffen J. Schmidt*; Viktoria S. Wurmbach*; Anette Lampert; Simone Bernard; Walter E. Haefeli; Hanna M. Seidling; Petra A. Thürmann (*both authors contributed equally to the work)

Corresponding author:
PD Dr. sc. hum. Hanna M. Seidling, University of Heidelberg, Department of Clinical Pharmacology and Pharmacoepidemiology, Cooperation Unit Clinical Pharmacy, Im Neuenheimer Feld 410, 69120 Heidelberg, Germany (E-mail: hanna.seidling@med.uni-heidelberg.de)
